# Effect of intraoperative mannitol administration on acute kidney injury after robot-assisted laparoscopic radical prostatectomy: A propensity score matching analysis

**DOI:** 10.1097/MD.0000000000011338

**Published:** 2018-06-29

**Authors:** Yu-Gyeong Kong, Ji Hyun Park, Jun-Young Park, Jihion Yu, Joonho Lee, Se-Ung Park, In Gab Jeong, Jai-Hyun Hwang, Hee Yeong Kim, Young-Kug Kim

**Affiliations:** a Department of Anesthesiology and Pain Medicine, Hangang Sacred Heart Hospital, Hallym University College of Medicine; b Department of Anesthesiology and Pain Medicine; c Department of Urology, Asan Medical Center, University of Ulsan College of Medicine, Seoul, Korea.

**Keywords:** acute kidney injury, mannitol, robot-assisted laparoscopic radical prostatectomy

## Abstract

Mannitol, an osmotic diuretic, has been used to prevent acute kidney injury (AKI). However, studies have found divergent effects of intraoperative mannitol administration on postoperative AKI. We therefore evaluated the effects of intraoperative mannitol administration on AKI after robot-assisted laparoscopic radical prostatectomy (RALP) in prostate cancer patients.

A total of 864 patients who underwent RALP were divided into mannitol (administered at 0.5 g/kg) and no-mannitol groups. Demographics, cancer-related data, preoperative laboratory values, intraoperative data, and postoperative outcomes such as AKI, chronic kidney disease at 12 months postoperation, duration of hospital stay, and intensive care unit admission rate and duration of stay were compared between the 2 groups using propensity score matching analysis. To determine the risk factors for AKI after RALP, univariate and multivariate logistic regression analyses were performed. Postoperative AKI was defined according to the Kidney Disease: Improving Global Outcomes criteria.

After performing 1:1 propensity score matching, the mannitol and no-mannitol groups included 234 patients each. The overall incidence of AKI after RALP was 5.1% and was not significantly different between the no-mannitol and mannitol groups in the propensity score-matched patients (13 [5.6%] vs. 11 [4.7%], *P* = .832). Univariate logistic regression analysis revealed that body mass index and operative time were associated with AKI in 864 patients who underwent RALP. However, intraoperative mannitol administration was not associated with AKI after RALP (*P* = .284). Multivariate logistic regression analysis revealed that operative time was significantly associated with AKI after RALP (odds ratio = 1.013, *P* = .001). The incidence of chronic kidney disease (13 [5.6%] vs. 12 [5.1%], *P* = 1.000) and other postoperative outcomes were not also significantly different between the no-mannitol and mannitol groups in the propensity score-matched patients.

Intraoperative mannitol administration has no beneficial effect on the prevention of AKI after RALP in prostate cancer patients. This result provides useful information for clinical practice guidelines regarding intraoperative mannitol use.

## 1 Introduction

Prostate cancer is the most common solid neoplasm and is the currently second-leading cause of cancer mortality for men.^[1]^ Radical prostatectomy is widely performed as a definitive treatment for localized prostate cancer.^[2]^ Robot-assisted laparoscopic radical prostatectomy (RALP) is widespread and currently accounts for the majority of radical prostatectomies.^[3]^ RALP is related to significantly fewer 30-day complications, lower blood loss and blood transfusion rates, lower anastomotic strictures, and shorter hospital stays than open retropubic radical prostatectomy.^[4]^ In addition, postoperative acute kidney injury (AKI) occurs at a lower incidence after RALP than after open retropubic radical prostatectomy.^[5]^ However, postoperative AKI is linked to increased morbidity, mortality, and health costs in hospitalized patients.^[6,7]^ Therefore, the prevention of postoperative AKI is important for improving the postoperative outcomes of robotic prostatectomy.

Diuretics are administered as a clinical strategy to prevent AKI as oliguria is a risk factor in affected patients,^[8]^ and diuretics are used to increase renal blood flow and urine output.^[9]^ Mannitol, an osmotic diuretic, has been used during surgery to prevent AKI because of its potentially renal-protective effects through increased renal blood flow, decreased intravascular cellular swelling, free radical scavenging, decreased renin production, and increasing intravascular volumes.^[10,11]^ However, the role of mannitol in the prevention of AKI has not been well established,^[12]^ and the results of previous studies of intraoperative mannitol administration for prevention or treatment of AKI have been conflicting.^[13–15]^ Although mannitol is known to be effective for preventing AKI in animal models^[16,17]^ as well as reducing the incidence of postoperative AKI in renal transplant patients,^[15,18]^ no beneficial effects of mannitol on the prevention of AKI in patients undergoing abdominal aortic or cardiac surgery have been described.^[13,14]^

We therefore evaluated the effects of intraoperative mannitol administration on postoperative AKI following RALP in prostate cancer patients. We compared the postoperative AKI incidence between patients who received intraoperative mannitol during RALP (mannitol group) or not (no-mannitol group) using propensity score matching analysis. We also compared postoperative outcomes, such as chronic kidney disease, duration of hospital stay, and intensive care unit admission rate and duration of stay between these 2 groups.

## 2 Methods

### 2.1 Patients

This was a single-center, retrospective, propensity score matching study of prostate cancer patients who underwent RALP between January 2015 and November 2016 at Asan Medical Center, Seoul, Republic of Korea. Ethical approval for this study was provided by the Institutional Review Board at Asan Medical Center, Seoul, Republic of Korea (Protocol No. 2017-1425). We excluded patients who had undergone any additional procedures with RALP, had a history of chronic kidney disease, had incomplete medical records, or who were converted to open surgery.

### 2.2 Anesthetic and surgical techniques

Anesthesia was induced using propofol and rocuronium and maintained using 1.5 to 2 vol% sevoflurane, a 50% oxygen/air mixture, remifentanil, and rocuronium. Mechanical ventilation was performed with a constant tidal volume of 8 mL/kg of ideal body weight and a respiratory rate of 10 to 18 cycles/min. The end-tidal carbon dioxide tension was maintained between 35 and 40 mm Hg during RALP. Continuous electrocardiography, heart rate, body temperature, and peripheral oxygen saturation were routinely monitored. In addition, arterial blood pressure was also monitored.

Fluid was administered according to our institutional protocol with a guidance of mean arterial blood pressure, heart rate, and blood loss. A crystalloid solution (Plasma solution A, CJ Pharmaceutical, Seoul, Korea) was administrated at a rate of 2 to 4 mL/kg/h. However, synthetic colloids, including 6% hydroxyethyl starch, were not administered during surgery. The additional fluid or vasoactive drugs such as ephedrine, phenylephrine, or norepinephrine were administered to patients if hypotension was observed (i.e., mean arterial blood pressure < 65 mm Hg). In addition, 30 minutes after surgical incision, mannitol (0.5 g/kg) was administered at the discretion of the treating physician.

RALP was performed in accordance with our institutional protocol.^[19,20]^ Briefly, pneumoperitoneum was established using a Veress needle, and 6 trocars were then inserted. Bladder mobilization permitted entry into the space of Retzius. The prostate was dissected via a transperitoneal antegrade approach. A nerve-sparing procedure was performed for all preoperatively potent patients on the sides not suspicious for cancer extension. A continuous suture was made during the vesicourethral anastomosis. The pelvic lymph nodes were selectively dissected in the low-risk group, and routinely dissected in the intermediate- to high-risk groups according to D’Amico criteria.^[21]^

### 2.3 Data collection and measurement

Electronic medical records were reviewed to collect baseline characteristics, cancer-related data, preoperative laboratory values, and intraoperative data on the study subjects. The baseline characteristics included age, body mass index, diabetes mellitus, hypertension, American Society of Anesthesiologists Physical Status classification, comorbidities such as cardiac, pulmonary, and cerebrovascular diseases, and the use of prescribed medications (beta blockers, nonsteroidal anti-inflammatory drugs and statins). Cardiac disease included coronary artery disease and heart failure. Coronary artery disease was defined as a history of ischemic heart disease diagnosed by a cardiologist, and heart failure was defined as a history of any type of heart failure that was diagnosed by a cardiologist with or without of medication or decreased ejection fraction (<40%). Cerebrovascular disease was defined as a history of carotid artery stent or angioplasty, transient ischemic attack, stroke, or cerebral hemorrhagic event.

Cancer-related data, including cancer stage, prostate-specific antigen level, and Gleason score, were also collected. Cancer stages were assigned in accordance with the 2010 American Joint Committee on Cancer tumor–node–metastasis staging system.^[22]^

Preoperative laboratory data included hemoglobin, platelet count, activated partial thromboplastin time, prothrombin time, albumin, uric acid, glucose, and estimated glomerular filtration rate (eGFR). The preoperative eGFR was calculated using the Chronic Kidney Disease Epidemiology Collaboration equation as follows: eGFR (mL/min/1.73 m^2^) = 141 × minimum (serum creatinine/k or 1)^α^ × maximum (serum creatinine/k or 1)^−1.209^ × 0.993^age^ × 1.018 (if female), where k is 0.7 for females and 0.9 for males, and α is −0.329 for females and −0.411 for males.^[23]^ Intraoperative data included the volume of administered crystalloid, use of vasopressors, and operative time.

### 2.4 Primary and secondary endpoints

The primary endpoint was the comparison of AKI incidence after RALP between the no-mannitol and mannitol groups of prostate cancer patients. Postoperative AKI was defined according to the Kidney Disease: Improving Global Outcomes criteria on the basis of postoperative changes in the serum creatinine levels and urine volume. AKI was defined as an increase in serum creatinine by ≥ 0.3 mg/dL within 48 hours postoperatively, an increase in serum creatinine to ≥ 1.5 times baseline within 7 days postoperatively, or an urine volume < 0.5 mL/kg/h for 6 hours postoperatively.^[24]^

The secondary endpoints included comparisons of the postoperative outcomes between the no-mannitol and mannitol groups. Postoperative outcomes included chronic kidney disease, duration of hospital stay, and intensive care unit admission rate and duration of stay. Chronic kidney disease was defined as an eGFR level of < 60 mL/min/1.73 m^2^.^[25]^ The hospital stay and intensive care unit stay durations were determined from the day after surgery, and the intensive care unit admission rate was calculated from the number of patients admitted to the intensive care unit after surgery.

### 2.5 Statistical analysis

Data are presented as means ± standard deviations, or numbers (percentages), as appropriate. Prior to propensity score matching, categorical variables were compared using the *χ*^2^ or the Fisher exact test, and continuous variables were compared using the *t* test or the Mann–Whitney *U* test. Because this was a retrospective observational study, patients were not randomized before it was decided whether to use mannitol. Hence, we performed a 1:1 propensity score matching analysis to reduce the influence of possible confounding variables and adjust the intergroup differences between the no-mannitol and mannitol groups. A multiple logistic regression model was created to calculate the propensity score and the following variables were tested for this: age, body mass index, diabetes mellitus, hypertension, American Society of Anesthesiologists Physical Status classification, cardiac disease, pulmonary disease, cerebrovascular diseases, beta blockers, nonsteroidal anti-inflammatory drugs, statins, cancer stage, prostate-specific antigen, Gleason score, hemoglobin, platelet count, activated partial thromboplastin time, prothrombin time, albumin, uric acid, glucose, eGFR, vasopressors, crystalloid, and operative time. The nearest available match between the 2 groups (no-mannitol and mannitol) according to propensity score similarities (caliper size of 0.2) was adopted. Standardized difference (difference in means between the 2 groups in units of standard deviation) was measured to evaluate balance between the 2 groups. Standardized difference value of < 0.2 was regarded as the cutoff for an adequate comparison. After 1:1 propensity score matching, continuous variables were compared using the paired *t* test or the Wilcoxon signed rank test, as appropriate, and categorical variables were compared using the McNemar test.

To determine the risk factors for AKI in 864 patients who underwent RALP, univariate logistic regression analysis was performed. Variables with a *P* value of < .1 in univariate logistic regression analysis were entered into multivariate logistic regression analysis with the forward stepwise conditional method. A *P* value of < .05 was considered statistically significant. All statistical analyses were conducted using SPSS for Windows version 22.0 (IBM-SPSS Inc, Armonk, NY).

## 3 Results

**Figure 1:**
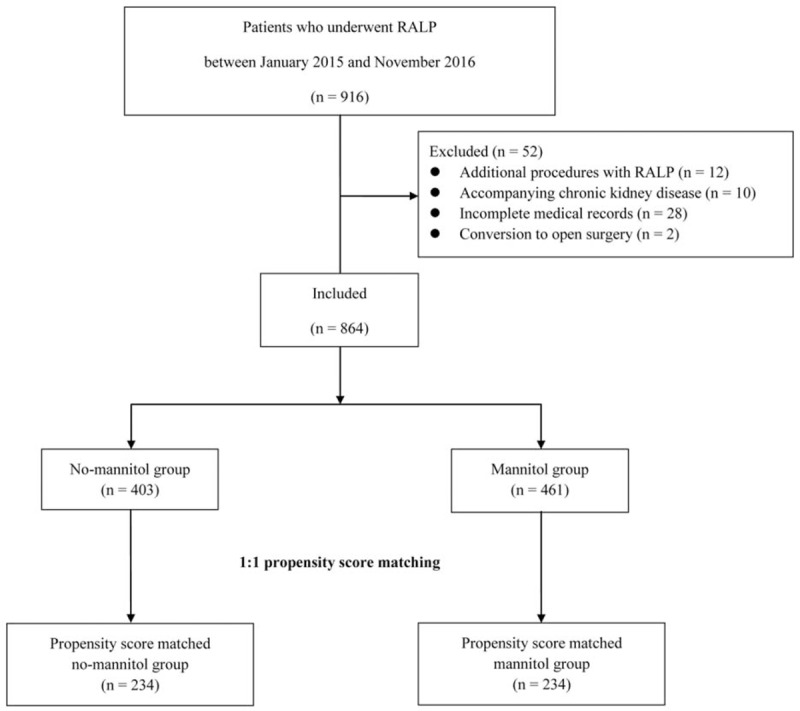
Study flow diagram. RALP = robot-assisted laparoscopic radical prostatectomy.

A medical chart review identified 916 prostate cancer patients who underwent RALP during the study period at our hospital. Of these cases, we excluded 52 patients due to additional procedures with RALP (n = 12), chronic kidney disease (n = 10), incomplete medical records (n = 28) and conversion to open surgery (n = 2). A total of 864 patients who underwent RALP without (n = 403) or with (n = 461) the administration of mannitol were included in the current study (Fig. 1).

We generated 234 matched pairs using the propensity score matching method with both study groups to counterpoise each variable. Hence, patients who underwent RALP were categorized into a no-mannitol (n = 234) or mannitol (0.5 g/kg during surgery; n = 234) group (Table 1). Because standardized differences were < 0.2 for all confounding variables, we deemed that our propensity score matching method was effective for balancing the 2 groups (Table 1).

**Table 1:**
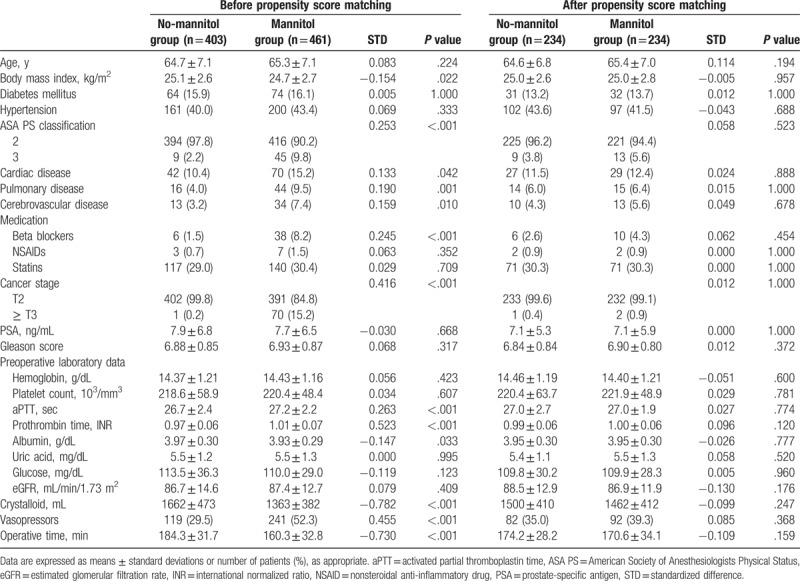
Demographic, cancer-related, preoperative, and intraoperative data for the no-mannitol and mannitol groups.

The demographic, cancer-related, preoperative, and intraoperative data for these patients are listed in Table 1. Prior to propensity score matching, statistically significant differences between the no-mannitol and mannitol patients were found for the body mass index, American Society of Anesthesiologists Physical Status classification, cardiac disease, pulmonary disease, cerebrovascular disease, beta-blocker use, cancer stage, activated partial thromboplastin time, prothrombin time, albumin, administered crystalloid, vasopressors, and operative time (Table 1). After performing 1:1 propensity score matching analysis however, there were no significant differences in demographic, cancer-related, preoperative, and intraoperative data between these 2 groups (Table 1).

Univariate logistic regression analysis revealed that body mass index and operative time were associated with AKI in 864 patients who underwent RALP (*P* <.1) (Table 2). However, intraoperative mannitol administration was not associated with AKI after RALP (*P* = .284). Multivariate logistic regression analysis revealed that operative time was significantly associated with AKI after RALP (odds ratio = 1.013, *P* = .001) (Table 2).

**Table 2:**
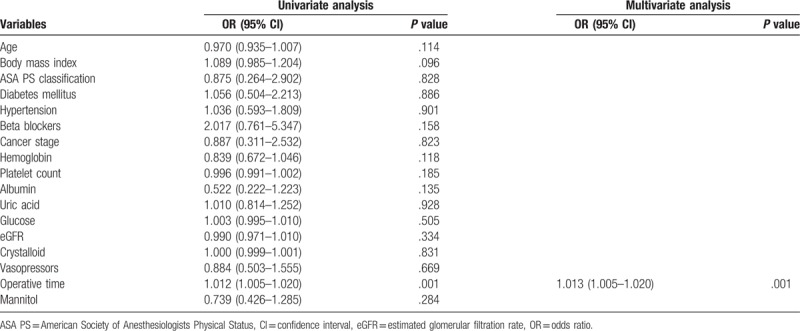
Univariate and multivariate logistic regression analyses for risk factors of acute kidney injury in 864 patients who underwent robot-assisted laparoscopic radical prostatectomy.

None of propensity score-matched patients underwent radiation therapy or chemotherapy prior to surgery. One patient in the no-mannitol group, but none in the mannitol group, underwent chemotherapy within 12 months of the surgery (0.4%) but this was not significant (*P* = 1.000). Twelve patients underwent radiation therapy within 12 months postsurgery but this was also not statistically different between the 2 groups (7 [3.0%] in the no-mannitol group vs. 5 [2.1%] in the mannitol group, *P* = .774).

**Figure 2:**
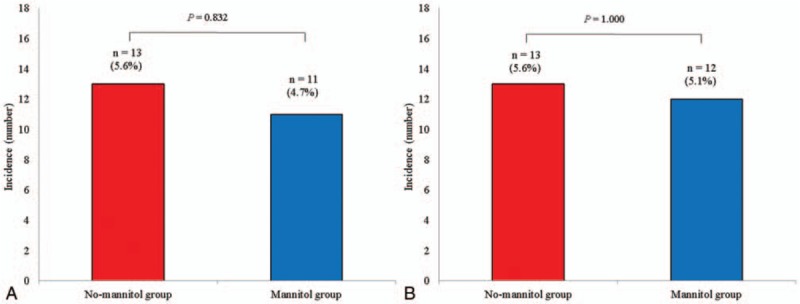
Comparison of the incidence of acute kidney injury (A) and chronic kidney disease (B) after robot-assisted laparoscopic radical prostatectomy between the no-mannitol and mannitol groups in the propensity score-matched patients. There were no significant differences in the incidence of postoperative acute kidney injury and chronic kidney disease between these 2 groups.

The postoperative outcomes in the propensity score-matched patients are presented in Table 3. The overall incidence of AKI after RALP was 5.1%. However, the incidence of postoperative AKI was not significantly different between the no-mannitol and mannitol groups (13 [5.6%] vs. 11 [4.7%], respectively; *P* = .832). The incidence of chronic kidney disease at 12 months postoperation was also not significantly different between the no-mannitol and mannitol groups (13 [5.6%] vs. 12 [5.1%], respectively; *P* = 1.000). Figure 2 shows a comparison of the incidence of postoperative AKI and chronic kidney disease between the 2 groups in the propensity score-matched patients. In addition, the duration of hospital stay and intensive care unit admission rate and duration of stay were not significantly different between the groups (Table 3).

**Table 3:**
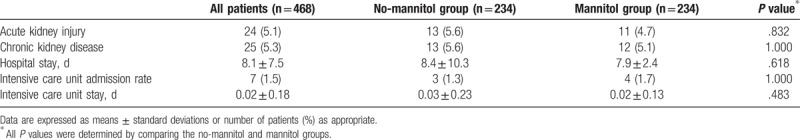
Postoperative outcomes in the propensity score-matched patients.

## 4 Discussion

We found from our current analysis that 5.1% of the prostate cancer patients who underwent RALP developed postoperative AKI, and that this incidence was not significantly different between patients who received intraoperative mannitol and those who did not. We also found that the incidence of chronic kidney disease at 12 months postsurgery was not significantly different between these 2 groups. We furthermore observed no significant differences in duration of hospital stay and intensive care unit admission rate and duration of stay between the 2 groups.

RALP has gained popularity as a new surgical treatment for localized prostate cancer and has many advantages such as lower blood transfusion rates and shorter hospital stays.^[3,4]^ However, prostate cancer patients are high-risk group for postoperative AKI because of their older age, tendency to develop obstructive uropathy, and higher risk of postoperative complications such as bleeding and urinary obstruction.^[1]^ In addition, RALP results in a decreased GFR, renal blood flow, and urine output due to pneumoperitoneum by carbon dioxide insufflation and the use of the steep Trendelenburg position during surgery.^[26–28]^ The overall incidence of AKI after RALP is reported to be approximately 5.5%.^[5]^ In our present study, the incidence of postoperative AKI in RALP was 5.1%. Postoperative AKI is defined as an abrupt decrease of renal function,^[29]^ and is associated with increased health costs, morbidity, and mortality.^[6,7]^

Many strategies have been proposed for preventing and treating AKI. Clinically, mannitol has been used as a renal protective agent in patients at high risk of developing renal injury. Mannitol induces a continued osmotic diuresis. This reduces ischemic injury through the accumulation of necrotic cell debris and hydroxyl and other free radicals due to the flushing effect. Mannitol also improves renal blood flow through a mechanism which reduces the production of renin and increases the production of vasodilating prostaglandin. However, there are also some detrimental overall effects of mannitol as the tubular reabsorption associated with increased GFR causes oxygen consumption.^[30]^ However, studies on the effects of mannitol administration on the renal function have produced conflicting findings, and there are no conclusive clinical data showing that mannitol can prevent postoperative AKI.

We found from our present investigation that intraoperative mannitol administration has no beneficial effect in the prevention of AKI after RALP. In line with our findings, several previous studies have reported no reduction in the incidence of renal failure in patients who underwent abdominal aortic aneurysm repair.^[14,31]^ In a study of elective open infra-renal abdominal aortic aneurysm repair, there were no significant differences in creatinine clearance, urinary albumin, and urinary creatinine from 2 hours to 7 days between the control group and the intervention group that received antioxidants including mannitol, with the exception of postoperative day 2.^[32]^ Smith et al previously reported findings in 2 studies on the effects of mannitol on renal function after cardiopulmonary bypass in patients with established renal dysfunction (creatinine 130–250 μmol/L)^[13]^ and those with preoperative normal creatinine level <130 μmol/L.^[33]^ These authors concluded that mannitol administration has no protective effect on renal function during cardiac surgery in patients with established renal dysfunction or in those with normal preoperative creatinine level. In addition, other reports have suggested that mannitol use does not influence renal function after nephrectomy.^[34–36]^ In a retrospective review of partial nephrectomy, mannitol administration had no effect on renal function recovery measured by the eGFR.^[36]^ Kenji et al^[34]^ also suggested that the administration of mannitol during open partial nephrectomy has no beneficial effects on the eGFR within 6 months of this surgery.

In contrast to the aforementioned clinical findings, earlier animal studies of mannitol administration reported an increase in the GFR^[37]^ and beneficial effects against contrast-induced nephropathy.^[38]^ In addition, it was further reported that mannitol infusion tends to move the GFR toward normal levels when given both before and after the induction of hypotension in hypoperfused animal kidneys.^[39,40]^ Furthermore, another study suggested that mannitol administration plus hydration offers a significant protection against renal function estimated by serum creatinine and cystatin-C 24 hours after endovascular aortic aneurysm repair compared to hydration alone.^[41]^ In that study, it was found that mannitol stimulates prostaglandin-I_2_ synthesis, increases renal blood flow, and reverses established renal vasoconstriction during endovascular aortic aneurysm repair. However, these protective effects were not maintained at 72 hours because renal dysfunction also is induced by various mechanisms, including renal microembolization and bilateral renal artery stenosis after the procedure. In another report, the effects of mannitol on renal function were evaluated in patients who underwent infrarenal aortic aneurysm repair.^[42]^ In that study, patients treated with mannitol had lower postoperative levels of urinary albumin and N-acetyl glucosaminidase, indicating a reduced level of subclinical glomerular and renal tubular damage, compared with those treated with saline. The authors in this case suggested that intraoperative mannitol administration reduces subclinical renal injury following infrarenal aortic aneurysm repair.

In several other studies however, mannitol administration has not only demonstrated ineffectiveness in terms of renal protection, but also the potential to cause renal failure.^[43,44]^ Forced euvolemic diuresis with mannitol was reported to increase the serum creatinine level and rates of contrast-induced nephropathy in patients with chronic kidney disease who underwent coronary angiography.^[43]^ According to various clinical reports therefore, the outcomes of mannitol use on renal function are either beneficial, ineffective, or deleterious. In our present study however, intraoperative mannitol administration had no impact on the incidence of postoperative AKI following RALP.

Our present study had some possible limitations. First, there were many potential confounders, such as age, body mass index, comorbidities, and preoperative laboratory examinations, which may have affected the accurate evaluation of the incidence of AKI after RALP. Although we performed propensity score matching analysis to minimize these biases, we cannot exclude the possibility of other confounders that may have affected the observed outcomes. Second, our patient cohort was derived from a single institution and our findings must be interpreted accordingly.

In conclusion, the incidence of postoperative AKI as well as postoperative outcomes such as chronic kidney disease, duration of hospital stay, and intensive care unit admission rate and duration of stay are unaffected by the use of mannitol in prostate cancer patients who have undergone RALP. These findings will contribute to future clinical practice guidelines for the intraoperative administration of mannitol during robotic prostatectomy.

## Acknowledgment

The authors thank Dr Joon Seo Lim from the Scientific Publications Team at Asan Medical Center for his editorial assistance in preparing this manuscript.

## Author contributions

**Conceptualization:** Hee Yeong Kim, Young-Kug Kim.

**Data curation:** Yu-Gyeong Kong, Jun-Young Park, Jihion Yu, Joonho Lee, Se-Ung Park, Young-Kug Kim.

**Formal analysis:** Yu-Gyeong Kong, Ji Hyun Park, Jun-Young Park, Jihion Yu, In Gab Jeong, Hee Yeong Kim, Young-Kug Kim.

**Investigation:** Yu-Gyeong Kong, Ji Hyun Park, Young-Kug Kim.

**Methodology:** Yu-Gyeong Kong, Ji Hyun Park, Jihion Yu, In Gab Jeong, Hee Yeong Kim, Young-Kug Kim.

**Project administration:** Young-Kug Kim.

**Supervision:** Jai-Hyun Hwang, Hee Yeong Kim, Young-Kug Kim.

**Validation:** Yu-Gyeong Kong, Ji Hyun Park, Hee Yeong Kim, Young-Kug Kim.

**Writing – original draft:** Yu-Gyeong Kong, Ji Hyun Park.

**Writing – review & editing:** Hee Yeong Kim, Young-Kug Kim.
